# Participation of *Candida albicans* Transcription Factor *RLM1* in Cell Wall Biogenesis and Virulence

**DOI:** 10.1371/journal.pone.0086270

**Published:** 2014-01-23

**Authors:** Yolanda Delgado-Silva, Catarina Vaz, Joana Carvalho-Pereira, Catarina Carneiro, Eugénia Nogueira, Alexandra Correia, Laura Carreto, Sónia Silva, Augusto Faustino, Célia Pais, Rui Oliveira, Paula Sampaio

**Affiliations:** 1 Centre of Molecular and Environmental Biology (CBMA), Department of Biology, University of Minho, Braga, Portugal; 2 Departamento de Imuno- Fisiologia e Farmacologia, ICBAS-UP - Instituto de Ciências Biomédicas de Abel Salazar, Universidade do Porto, Porto, Portugal; 3 RNA Biology Laboratory, Department of Biology and CESAM, University of Aveiro, Portugal; 4 Institute for Biotechnology and Bioengineering, Centre of Biological Engineering, University of Minho, Braga, Portugal; 5 Departamento de Patologia e Imunologia Molecular, ICBAS- UP, Instituto de Ciências Biomédicas de Abel Salazar, Universidade do Porto, Porto, Portugal; The Ohio State University, United States of America

## Abstract

*Candida albicans* cell wall is important for growth and interaction with the environment. *RLM1* is one of the putative transcription factors involved in the cell wall integrity pathway, which plays an important role in the maintenance of the cell wall integrity. In this work we investigated the involvement of *RLM1* in the cell wall biogenesis and in virulence. Newly constructed *C. albicans Δ/Δrlm1* mutants showed typical cell wall weakening phenotypes, such as hypersensitivity to Congo Red, Calcofluor White, and caspofungin (phenotype reverted in the presence of sorbitol), confirming the involvement of *RLM1* in the cell wall integrity. Additionally, the cell wall of *C. albicans Δ/Δrlm1* showed a significant increase in chitin (213%) and reduction in mannans (60%), in comparison with the wild-type, results that are consistent with cell wall remodelling. Microarray analysis in the absence of any stress showed that deletion of *RLM1* in *C. albicans* significantly down-regulated genes involved in carbohydrate catabolism such as *DAK2*, *GLK4*, *NHT1* and *TPS1*, up-regulated genes involved in the utilization of alternative carbon sources, like *AGP2*, *SOU1*, *SAP6*, *CIT1* or *GAL4*, and genes involved in cell adhesion like *ECE1*, *ALS1*, *ALS3*, *HWP1* or *RBT1*. In agreement with the microarray results adhesion assays showed an increased amount of adhering cells and total biomass in the mutant strain, in comparison with the wild-type. *C. albicans* mutant *Δ/Δrlm1* strain was also found to be less virulent than the wild-type and complemented strains in the murine model of disseminated candidiasis. Overall, we showed that in the absence of *RLM1* the modifications in the cell wall composition alter yeast interaction with the environment, with consequences in adhesion ability and virulence. The gene expression findings suggest that this gene participates in the cell wall biogenesis, with the mutant rearranging its metabolic pathways to allow the use of alternative carbon sources.

## Introduction

The yeast cell wall is an essential cellular structure for the osmotic stabilization, protection against mechanical damage, maintenance of cell shape, adhesion, and invasive growth [1]. It consists of a matrix of β-glucan, chitin and mannoproteins, surrounding the plasma membrane. β-glucan is the major constituent of the cell wall inner layer and is responsible for the shape of the cell. Chitin, a polymer required during bud-site selection and septation is localized next to the plasma membrane and is responsible for the wall rigidity. The cell wall outer layer is involved in many interactions with the environment and is formed by cell wall proteins, which are often highly mannosylated [2]. The cell wall is a dynamic structure since it changes with alterations of the surrounding growth conditions [3] and is remodeled as the cell increases in size and during morphogenetic processes such as mating, sporulation, or pseudohyphal growth. Upon cell wall damage, cells activate the cell wall integrity (CWI) mitogen-activated protein (MAP) kinase pathway (also known as the PKC pathway) so that the cell wall is repaired and cell integrity maintained. This response involves several processes: (i) the balance between cell wall polysaccharides is modified, as indicated by hyper-accumulation of chitin; (ii) the type of association between β-glucan, mannoproteins, and chitin is changed; (iii) an increase of cell wall proteins occurs; and (iv) the β-1,3-glucan synthase complex is transiently redistributed throughout the cell [3], [4]. The response to cell wall damage is well understood from studies with the budding yeast *Saccharomyces cerevisiae*, where the PKC-MAPK pathway is the major cell wall responsive regulatory system not only in stress conditions but also during cell wall biogenesis. This pathway comprises the sensors Mid2 and Wsc1 in the plasma membrane which, upon cell wall damage, interact with the GDP/GTP exchange factor Rom2, leading to the conversion of the G protein Rho1 to its activated GTP-bound state. Interaction with Rho1 activates the control kinase Pkc1 that phosphorylates Bck1, the first component of the MAP kinase cascade three-component module. Sequential phosphorylations of the components of the MAP kinase cascade activate downstream kinases: the redundant Mkk1 and Mkk2 and the final kinase Slt2. The targets of the PKC-MAPK pathway are the transcription factor heterodimer complex SBF (composed by Swi4 and Swi6), and the MADS-box transcription factor Rlm1 [5], [6], which are the effectors of the pathway. In accordance with the complexity of the cellular processes related to cell wall homeostasis in yeast, cross-talk between distinct MAPK pathways has recently been described [2]. The calcineurin and high-osmolarity pathways have been shown to participate in the response to cell wall damages.

As an opportunistic pathogen, *Candida albicans* is able to adapt its growth to a range of environmental changes by modulation of expression of many genes in a coordinated manner. Many of the MAP kinase pathway components are important for virulence and morphological transitions [7]–[9]. In *C. albicans* Mkc1, the homologue of the *S. cerevisiae* Slt2/Mpk1 MAPK, mediates PKC-MAPK pathway [10]–[13]. The kinase Mkc1 becomes activated in response to several types of stress such as oxidative, osmotic, cell wall damage, calcium ions and temperature [12], [14], [15]. Mutants affected in *MKC1* are more sensitive to cell wall degrading enzymes and antifungals, and display surface alterations when grown under restrictive conditions such as high temperature [10], [11]. Not surprisingly, mkc1 mutants display a reduced virulence in the mouse model of systemic infection [13]. Several components of the PKC-MAPK pathway were identified based on sequence and functional homologies with *S. cerevisiae* and their involvement in the cell wall regulation revealed a broader action than their *S. cerevisiae* orthologs [10], [16]. Targets of Mkc1 (*SWI4*, *SWI6* and *RLM1*) have been identified in *C. albicans* genome by sequence homology with *S. cerevisiae*, suggesting that these could act as final effectors of the signaling cascade in the pathogenic yeast. However, although *C. albicans RLM1* was required for normal growth in the presence of caspofungin and Congo Red, this gene functions were not essential for the transcriptional response to caspofungin, suggesting that it is required more generally for cell wall structure or integrity [14]. In contrast, a zinc finger protein, Cas5, was identified as being required for expression of numerous caspofungin-responsive genes. It was then suggested that *C. albicans* Cas5 may be the functional equivalent of *S. cerevisiae* Rlm1 and implicated in the response to cell wall damage.

The main objectives of this work were to determine the involvement of *CaRLM1* in cell wall biogenesis, and evaluate the consequence of its absence in *C albicans* virulence. These studies were performed by using a set of *rlm1* mutants constructed with the *SAT1*-flipping strategy [17]. *Candida albicans rlm1* mutant significantly down-regulated genes involved in carbohydrate catabolism such as *DAK2*, *GLK4*, *NHT1* and *TPS1* and up-regulated genes involved in the utilization of alternative carbon sources, like *AGP2*, *SOU1*, *SAP6*, *CIT1* or *GAL4*, which suggests the involvement of CaRlm1 in cell wall biogenesis, particularly in regulating the flow of carbohydrates into cell wall biosynthesis pathways. Additionally, the modifications in cell wall composition of this mutant, and significant up-regulation of genes involved in cell adhesion, like *ECE1*, *ALS1*, *ALS3*, *HWP1* or *RBT1*, altered the yeast interaction with the environment, with consequences for the adhesion ability as well as for virulence in the mice model of disseminated candidiasis.

## Materials and Methods

### Strains and growth conditions

*Candida albicans* and *S. cerevisiae* strains used in this study are listed in Table S1. All strains were stored as frozen stocks with 15% (w/v) glycerol at −80°C and cultured on yeast extract-peptone-dextrose (YPD) agar plates (1% w/v yeast extract, 2% w/v peptone, 2% w/v dextrose and 2% w/v agar) at 30°C. For routine growth of the strains, YPD liquid medium was used. Selection of *C. albicans* nourseothricin-resistant (Nou^R^) transformants was performed on YPD agar plates containing 200 µg/ml nourseothricin (Werner Bioagents, Jena, Germany). To obtain nourseothricin-sensitive (Nou^S^) derivatives in which the *SAT1* flipper was excised by FLP-mediated recombination, transformants were grown overnight in YCB–BSA–YE medium (2.34% w/v yeast carbon base, 0.4% w/v bovine serum albumin, 0.2% w/v yeast extract, pH 4.0) without selective pressure to induce the *SAP2* promoter controlling *caFLP* expression. One hundred to two hundred cells were then spread on YPD plates containing 10 µg/ml nourseothricin and incubated for 2 days at 30°C. Nou^S^ clones were identified by their small colony size and confirmed by re-streaking on YPD plates containing 200 µg/ml nourseothricin as described previously [17].

### Plasmid construction

The *RLM1* deletion construct was generated as follows: 0.5 kb of upstream and downstream flanking sequences were amplified from the genomic DNA of strain SC5314 with the primers pairs RLM1-1/RLM1-2 and RLM1-3/RLM1-4, respectively (Table S2), and the SacI/SacII- and XhoI/ApaI-digested PCR products were cloned on both sides of the *SAT1* flipper cassette of pSFS5 [18] to generate pRLM1M1. For reintroduction of *RLM1* into *rlm1* mutants, the *RLM1* coding region and ca. 0.5 kb of upstream and ca. 1.0 Kb downstream sequences were amplified with the primers RLM1-1 and RLM1-compl (Table S2). The PCR product was digested with SacI and SacII and used to replace the *RLM1* upstream region in pRLM1M1, resulting in pRLM1K1A.

### *Candida albicans* transformation

Strains of *C. albicans* were transformed by electroporation [19] with gel-purified SacI-ApaI fragments from pRLM1M1 were used to delete the *RLM1* gene, in strains SC5314 and 124a. The SacI-ApaI fragment from pRLM1K1A was used to reintroduce a functional *RLM1* copy into *rlm1* mutants. Nourseothricin-resistant transformants were selected on YPD agar plates containing 200 µg/ml nourseothricin as described previously [17]. The correct genomic integration of all constructs was confirmed by Southern hybridization with gene-specific probes.

### Southern hybridization

Genomic DNA from *C. albicans* strains was isolated as described previously [20]. Ten mg of DNA were digested with *EcoR*I, separated in a 1% (w/v) agarose gel and, after ethidium bromide staining, transferred by vacuum blotting onto a nylon membrane and fixed by UV crosslinking. The gel-purified *Sac*I-*Sac*II *RLM1* upstream fragment and *Xho*I-*Apa*I *RLM1* downstream fragment from pRLM1M1 were used as probes. Southern hybridization with enhanced chemiluminescence-labeled probes was performed with the Amersham ECL™ Direct Nucleic Acid Labelling and Detection System (GE Healthcare, Braunschweig, Germany) according to the instructions of the manufacturer.

### Susceptibility assays

Cultures were incubated overnight in liquid YPD medium at 30°C, 200 rpm, and diluted to OD_640_ = 1 with fresh medium. Drop tests were performed by spotting 5 µl of the serially diluted cell suspension onto 20% YPD (0.4% w/v peptone, 0.4% w/v glucose, 0.2% w/v yeast extract and 2% w/v agar) and YPD plates supplemented with the following compounds: 70 µg/ml calcofluor white (CFW), 100 µg/ml Congo Red (CR), 30 ng/ml caspofungin (CFG), 10 mM caffeine, 0.035% (w/v) SDS, 1.5M NaCl or 2M sorbitol. Plates were incubated 48 h at 30°C before observation. Nitrogen starvation sensitivity on solid media was assayed by growing cells on a YCB plate for more 6 days at 30°C and then blot into YPD [21].

### Filamentation tests

Cultures of *C. albicans* cells were grown for 24 h on modified Lee medium (0.5% w/v (NH_4_)_2_SO_4_, 0.02% w/v MgSO_4_.7H_2_O, 0.25% w/v K_2_HPO_4_, 0.5% w/v NaCl, 1.25% w/v D-galactose, 0.05% w/v L-alanine, 0.13% w/v L-leucine, 0.1% w/v L-lysine, 0.01% w/v L-methionine, 0.007% w/v L-ornithine, 0.05% w/v L-proline, 0.05% w/v L-threonine and 0.0001% w/v biotin, pH 5.0) at 26°C and 150 rpm in order to maintain cells in the yeast morphology. Filamentation was induced by plating approximately 30 colony-forming units in Spider medium (1% w/v nutrient broth, 1% w/v mannitol, 0.2% w/v K_2_HPO_4_, 1.35% w/v Bacto Agar, pH 7.2) or YPD supplemented with 10% (v/v) fetal bovine serum (FBS) medium. Plates were incubated at 37°C for 72 h and photographed.

### Quantification of cell wall components

The different cell wall sugar polymers were quantified in cells exponentially grown in YPD liquid medium at 30°C, by HPLC, following chemical hydrolysis with concentrated sulphuric acid, as previously described [22].

### DNA microarrays analysis

#### RNA isolation and sample labeling

*C. albicans* yeast cells from the wild-type SC5314 and Δ/Δ*rlm1* mutant strain were inoculated into 10 ml of YPD and grown overnight at 30 °C. Each overnight culture was used to inoculate 20 ml of YPD to an initial OD640 of 0.4, and incubated at 30 °C for an additional 2 hours at 150 rpm. The cells were then harvested and immediately stored at −80 °C. RNA extraction was performed by using the hot acidic phenol method [23]. cDNA synthesis and labeling were carried out as described elsewhere [24]. Briefly, cDNA was synthesized from 40 µg of total RNA in the presence of 2-aminoallyl-dUTP. Samples were purified using Microcon-30 (Millipore) columns prior to coupling to NHS ester activated Cy3 and Cy5 fluorofores. Before hybridization, free dyes were removed using Chromaspin-30 (Clontech) columns and the efficiency of cDNA synthesis and dye incorporation was measured by spectrophotometry (NanoDrop). All samples had a degree of labeling (labeled nucleotides per 100 nucleotides) of around 5.0±1.5.

#### DNA microarrays

Samples were hybridized onto customized Agilent 44K microarrays with probes designed for the *C. albicans* (Assembly 21) genome sequence. The microarray design was developed by the group of Prof. Geraldine Buttler at the School of Biomolecular and Biomedical Science, Conway Institute, University College, Dublin, and was made available upon request http://www.ucd.ie/biochem/gb/Lab/. The hybridizations were conducted following Agilent Technologies recommendations. Two independent microarray hybridizations for the comparison of SC5314 and Δ/Δ*rlm1* strains were carried out using dye-swap labeling. Microarray images were obtained at a 5 µm resolution using the Agilent G2565AA scanner. Fluorescence intensity was measured using the Agilent Feature Extraction Software (version 10.5.1.1) and signal and background quantitation was performed according to protocol Agilent recommendations (GE2_105_Dec08). Local background subtraction, Lowess normalization and averaging of replicate probes were performed using BRB Array Tools 3.8.0 http://linus.nci.nih.gov/BRB-ArrayTools.html. The processed signal was annotated using the data downloaded from the Candida Genome Database http://www.candidagenome.org/.

#### Statistical analysis and functional annotation of the data

The log2 intensity ratios were used for identification of differentially expressed genes, using the one class t-test implemented in TM4 Microarray SoftwareSuite (MeV) v4.6.1 and a *P*-value cut-off of 0.05. Only genes with a fold variation above 2 were considered for discussion. The web based tool Genecodis2 http://genecodis.dacya.ucm.es/ was used for functional enrichment analysis of the differentially expressed genes. *P*-values were calculated using the hypergeometric distribution and were corrected using the simulation-based approach [25]. Enrichment results were filtered using a corrected *P*-value cut-off of 0.05.

In order to identify the potential targets for CaRlm1p the YEASTRACT software (http://www.yeastract.com/), developed for *S. cerevisiae*, was used to search *C. albicans* upstream sequences. In this analysis we used all consensus nucleotide sequences reported in the literature as potential binding sites for ScRlm1p: ATAAATATAGA, CCTAAAAATAGA, CTAWWWWTAG, TAWWWWTAGM, TCTATTTCTAT, TCTATTTTTAC and TTATTTTTAGA, and assumed that they were conserved in *C. albicans* [26]–[28]. Since Lenardon *et al.* [28] reported the presence of Rlm1p functional sequences in a region ranging from the ATG start codon to −429 bp of the promoter region of *C. albicans* chitin synthase encoding genes, we established a region of 1000 bp from the promoter region of each identified ORF to search for potential Rlm1p target sequences.

#### RT-PCR

To validate microarray data, the transcript levels of some selected genes (*ALS3*, *HWP1*, *AGP2*, *PUT2*, *GCV2*, and *CIT1*) were also determined using a quantitative RT-PCR approach. Oligonucleotides used for this analysis are listed in Table S2. Total RNA was incubated with RNAse-free DNAase I (Invitrogen), for 15 minutes at room temperature to eliminate genomic DNA contamination. DNase was inactivated according to manufacturer's instructions. The Superscript III Platinium two-step qRT-PCR with SYBR green (Invitrogen) was used to generate first strand c-DNA from each DNase I treated RNA sample, as follows: 10 min at 25°C, 50 min at 42°C and 5 min at 85°C and then incubated 20 min at 37°C for RNase H treatment. Two microliters of each cDNA sample was added to a 20 µl PCR mixture containing 10 µl of Platinum SYBR green qPCR SuperMix-UDG, 0.4 µl of 10 µM specific forward and reverse primers and 7.2 µl of RNase-free water. Each reaction was performed with a Real Time PCR detection system (BIO-RAD). Thermocycling conditions for qPCR were 2 min at 50°C (UDG incubation) and 2 min at 95°C, followed by 45 cycles of 95°C for 15 s, 60°C for 30 s, and 72°C for 30 s. The specificity of each primer pair was verified by the presence of a single melting temperature peak. The efficiency of the primers was assessed in titration experiments using cDNA in serial dilutions. A negative (water) control and a four-point curve of SC5314 genomic DNA were included in each run. Gene expression was normalized to the housekeeping gene *ACT1* and analyzed by using the comparative threshold cycle (ΔΔCT) method. Data was presented as the fold difference in expression relative to wild-type (WT) gene expression.

#### Adhesion assay

Adhesion was assessed through quantification of total biomass by crystal violet (CV) staining [29], [30]. For this, standardized cell suspensions (1 ml containing 1×10^7^ cells ml^−1^ in YPD) were placed into selected wells on 12 polystyrene plates (Orange Scientific, Braine-l'Alleud, Belgium) and incubated at 37°C in a shaker at 120 rpm. Adhesion ability was measured after 2 h, 24 h and 48 h of incubation. Regarding the 48 h sample, an extra step was performed, at 24 h, 500 µl of YPD medium were removed and an equal volume of fresh YPD added. After the defined times of incubation, the medium was aspirated and non-adherent cells removed by washing the wells with sterile ultra-pure water. Regarding total biomass quantification the cells were fixed with 1 ml of methanol, which was removed after 15 min contact. The plates were allowed to dry at room temperature, and 1 ml of CV (1% v/v) added to each well and incubated for 5 min. The wells were then gently washed with sterile, ultra-pure water and 1 ml of acetic acid (33% v/v) was added to release and dissolve the stain. The absorbance of the obtained solution was read in triplicate in a microtiter plate reader (Bio-Tek Synergy HT, Izasa, Lisbon, Portugal) at 570 nm. Results were presented as absorbance/area of the wells (abs/cm^2^). Experiments were repeated in three independent assays.

### *Candida albicans* hematological disseminated infections

#### Ethics Statement

This study was carried out in strict accordance with the recommendations of the European Convention for the Protection of Vertebrate Animals used for Experimental and Other Scientific Purposes (ETS 123) and 86/609/EEC Directive and Portuguese rules (DL 129/92). The animal experimental protocol was approved by the competent national authority, Direcção Geral de Veterinária (DGV) (Protocol Permit Number: 0420/000/000/2008). All animal experiments were planned in order to minimize mice suffering.

#### Mice

Female BALB/c mice, 8 weeks old, were purchased from Charles River (Barcelona, Spain) and kept under specific-pathogen-free conditions at the Animal Facility of the Instituto de Ciências Biomédicas Abel Salazar, Porto, Portugal.

#### Yeast inoculum preparation

To prepare the inocula for mice infection *C. albicans* strains were grown in a shaking incubator for 14 h at 30°C in Winge medium (0.2% w/v glucose, 0.3% w/v yeast extract). Then yeast cells were harvested, washed twice with sterile, nonpyrogenic phosphate buffered saline (PBS), counted in a hemocytometer, and resuspended at 2.5×10^6^ cells/ml. Inocula were confirmed by CFU counts on YPD agar after 48 h at 37°C.

#### *Candida albicans* infection

Mice (n = 8/group) were injected intravenously (i.v.) in the lateral tail vein with 5×10^5^ *C. albicans* yeast cells in 0.2 ml PBS. To evaluate the progress of hematogenously disseminated candidiasis, mice were weighed and monitored twice per day. Mice that displayed severe signs of illness and/or showed severe weigh loss were euthanized immediately. Moribund mice were humanely terminated by placing them in a closed chamber filled with CO_2_ and their deaths were recorded as occurring on the following day. To analyze organ fungal burden, histology and gene expression, groups of mice (n = 4/group) were infected with the same inocula, and sacrificed 2 and 7 days post-infection, as previously described. Control mice were injected i.v. with PBS. After infection, kidneys were aseptically removed, weighed, homogenized, and quantitatively cultured on YPD agar at 37°C. Values were expressed as log CFU per gram of tissue. Alternatively, the other kidney, the liver and spleen were fixed in 10% phosphate buffered formaldehyde, followed by periodic acid-Schiff (PAS) reagent staining and counterstaining of the paraffin-embedded tissues with hematoxylin in order to evaluate both fungal morphology and composition, and distribution of inflammatory infiltrates.

### Statistical Analysis

Results were compared using a one-way analysis of variance (ANOVA) by applying Levene's test of homogeneity of variance and the Bonferroni's multiple-comparisons test, by using the GraphPad Prism 4 Software (GraphPad Software, Inc., La Jolla, CA, USA) or SPSS software (SPSS [Statistical Package for the Social Sciences], Inc., Chicago, IL). Results were considered statistically significant with P values of less than 0.05.

## Results

### *Candida albicans rlm1* mutant is hypersensitive to cell wall perturbing agents

**Figure 1:**
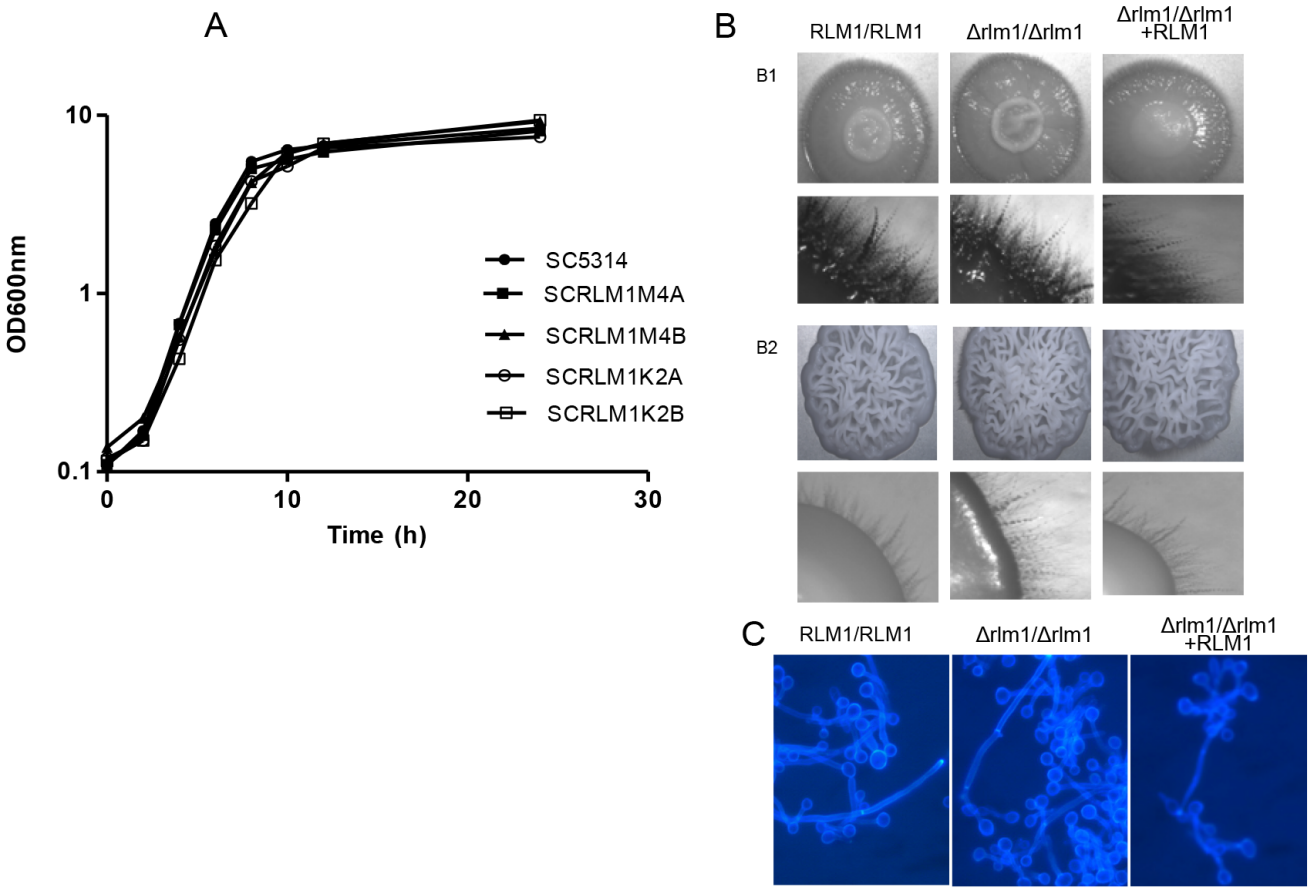
Growth and filamentation of *RLM1* wild-type, mutant, and complemented strains. (A) Growth curve of *C. albicans* SC5314 (RLM1/RLM1), homozygous mutants SCRLM1M4A and SCRLM1M4B (*Δrlm1/Δrlm1*) and complemented SCRLM1K2A and SCRLM1K2B (*Δrlm1/Δrlm1+ RLM1*) strains in YPD liquid medium at 30°C and (B) morphology of the colonies on Spider medium (B1) and on YPD containing 10% serum (B2) after 6 days of incubation at 30°C. Photographs of the colony edge are also presented. (C) Morphology of calcofluor white stained yeast cells grown for 5 hours on YPD with 10% serum.

With the objective of determining the role of *CaRLM1* in the cell wall biogenesis and its involvement in the virulence of this pathogenic species, a new set of *rlm1* null mutants was constructed from the prototrophic wild-type model strain SC5314, by using the *SAT1*-flipping strategy [17] to avoid the use of auxotrophic markers. Two independent mutant strains (SCRLM1M4A and B), in which both *RLM1* alleles were deleted, and two complemented strains (SCRLM1K2A and B) were generated after reintegration of *RLM1* ORF (Table S1). These two independently generated mutant strains were used to guarantee that the results obtained were caused by *RLM1* gene deletion and not due to any defects resultant from mutant construction. Before the phenotypic tests, growth of the constructed strains was assessed in YPD liquid medium at 30°C and 37°C, as well as their ability to filament on inducing media. Results showed no difference between the strains ability to grow at both temperatures and to filament (Figure 1 and data not shown).

**Figure 2:**
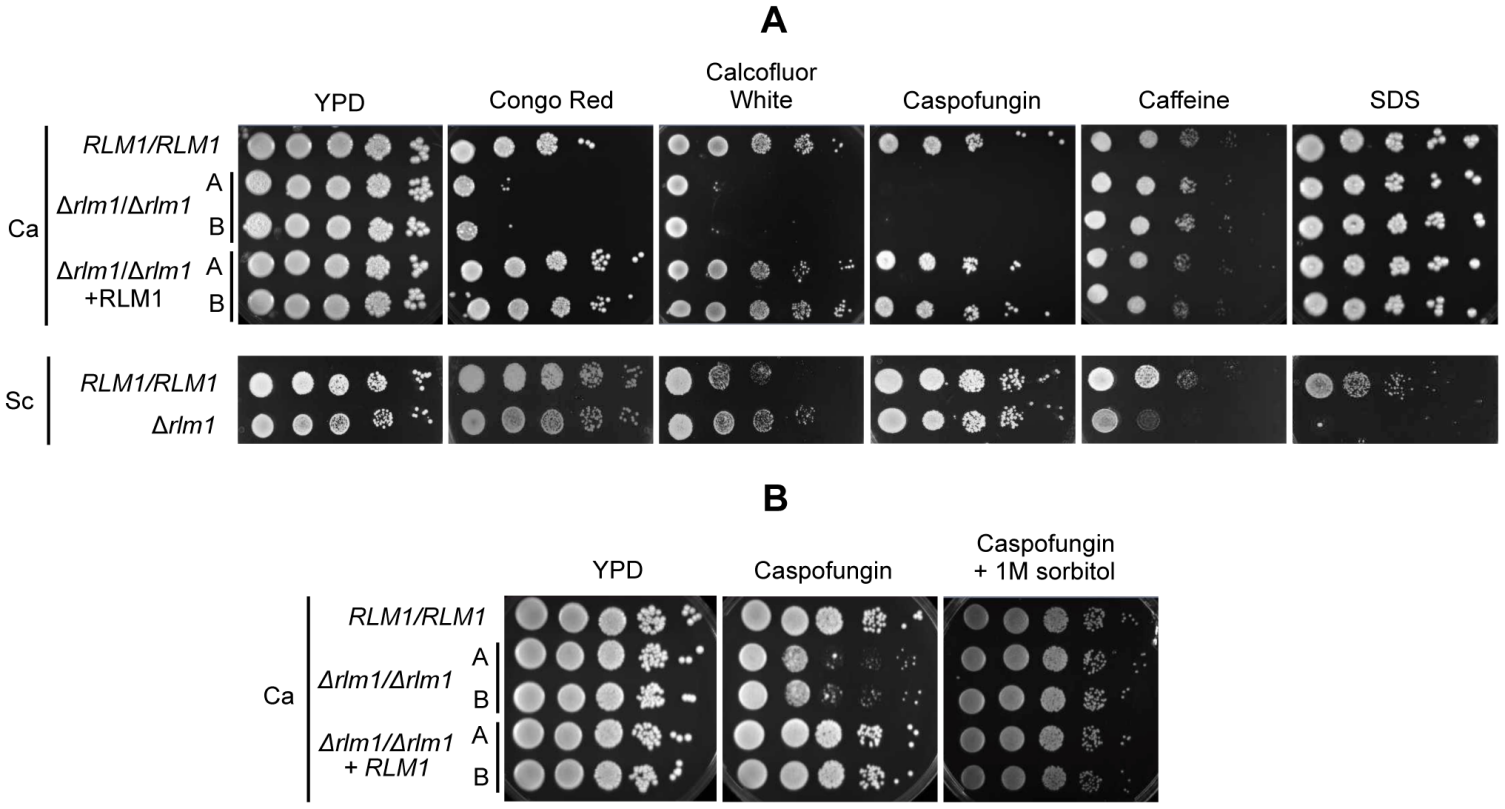
Growth of *C. albicans* wild-type, mutant and complemented strain, and *S. cerevisiae* wild-type and mutants in medium with several stress agents. (A) Serial 10-fold dilutions of YPD overnight cultures of the strains were spotted on YPD plates without or with 100 µg/ml congo red, 70 µg/ml calcofluor white, 30 ng/ml caspofungin, 10 mM caffeine or 0.0035% SDS and plates incubated for 2 days at 30°C. The *C. albicans* wild-type SC5314 strain (RLM1/RLM1), two independent homozygous *rlm1* mutants strains SCRLM1M4A and SCRLM1M4B (*Δrlm1/Δrlm1*), and two complemented strains SCRLM1K2A and SCRLM1K2B (*Δrlm1/Δrlm1+ RLM1*), as well as the *S. cerevisiae* haploid wild-type BY4741 (*RLM1*) and rlm1 mutant (*Δrlm1*) strains are shown. (B) Effect of the protection of 1M sorbitol in cells treated with Caspofungin. The *C. albicans* wild-type SC5314 strain (*RLM1/RLM1*), two independent homozygous *rlm1* mutants strains SCRLM1M4A and SCRLM1M4B (*Δrlm1/Δrlm1*), and two complemented strains SCRLM1K2A and SCRLM1K2B (*Δrlm1/Δrlm1+ RLM1*) are shown.

In order to evaluate if the strains constructed in this study also confer cell wall typical phenotypes, *C. albicans* strains were tested along with the *S. cerevisiae Δrlm1* mutant (YPL089c), for comparison. *C. albicans Δrlm1/Δrlm1*, as well as *S. cerevisiae Δrlm1* mutants were able to resist nitrogen starvation, grow at elevated temperatures and on glycerol medium (data not shown). In contrast, *S. cerevisiae Δrlm1* was sensitive to caffeine, while *C. albicans Δrlm1/Δrlm1* was able to grow under this condition (Figure 2A). Since *C. albicans RLM1* mutants did not show any of the phenotypes described for *S. cerevisiae* CWI mutants, the sensitivity of the deleted strains to a range of cell wall-perturbing agents, as well as to compounds known to be associated with altered cell walls, was determined. The absence of a functional *RLM1* in *C. albicans* resulted in hypersensitivity to Congo Red (CR), Calcofluor White (CFW) and Caspofungin (CFG), confirming previous results [14]. On the contrary, *S. cerevisiae Δrlm1* mutant showed unaffected growth in the presence of CR and CFG and displayed slight higher resistance to CFW, confirming results reported by Dodou and Treisman [31] and Garcia *et al.* [3]. The hypersensitivity of *C. albicans* mutant strains to CFG was reverted with the osmotic protection of 1M sorbitol (Fig. 2B)..However, growth of *C. albicans Δrlm1/Δrlm1* strains in the presence of SDS was unaffected, while for *S. cerevisiae Δrlm1* this compound caused decreased growth.

**Figure 3:**
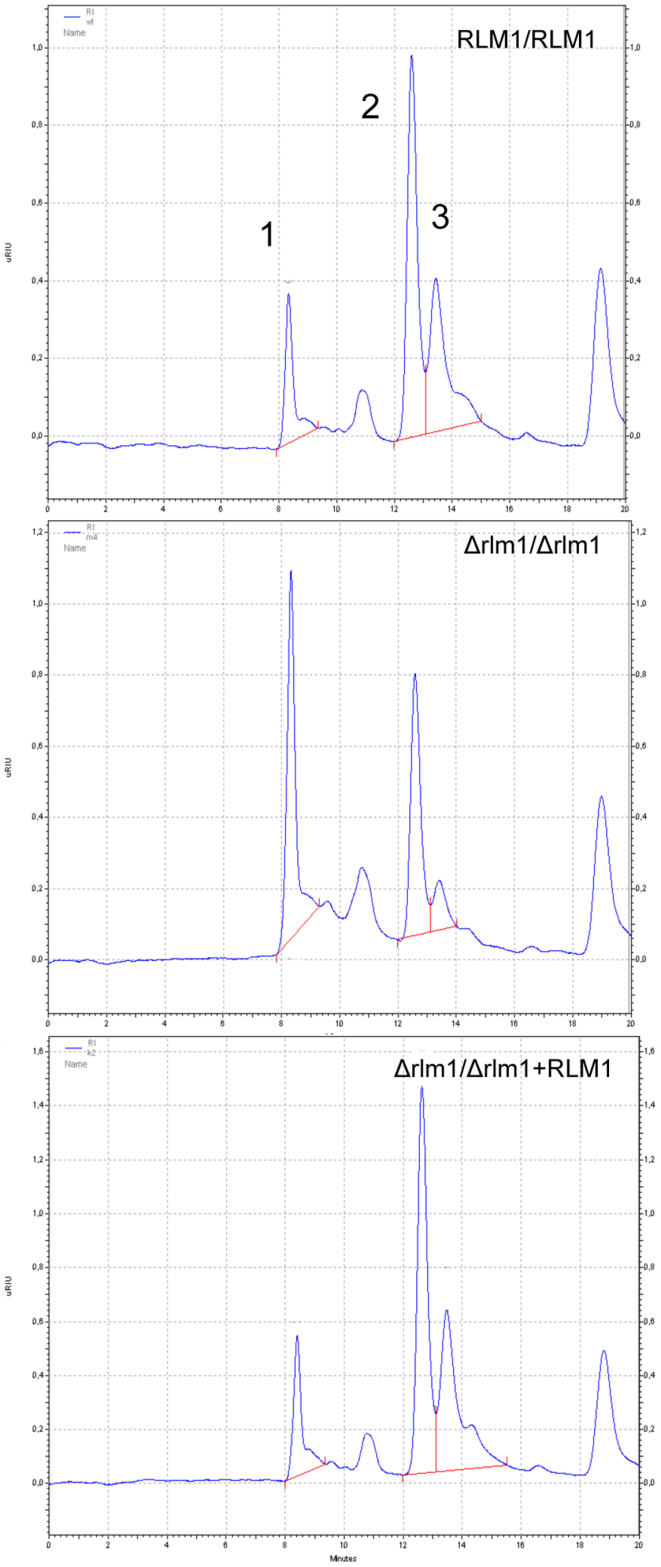
HPLC patterns of *C. albicans* wild-type, mutant and complemented cell wall sugars. Representative HPLC chromatogram of the separation of the main cell wall sugars, (1-glucosamine, 2-glucose and 3-mannose), after sulphuric acid hydrolysis of the cell wall from *C. albicans* wild-type (*RLM1/RLM1*), *rlm1* mutant *Δrlm1/Δrlm1* (SCRLM1M4A), and complemented *Δrlm1/Δrlm1+ RLM1* (SCRLM1K2A) strains.

Results from the phenotypic characterization suggested changes in the cell wall of *C. albicans* mutant cells. Therefore, the amounts of the different cell wall sugar polymers in *C. albicans* cells exponentially growing in YPD liquid medium were quantified by HPLC after sulphuric acid hydrolysis of the cell walls. The cell wall sugar composition of *C. albicans* null mutant, expressed as a percentage of the wild-type levels, was 60% (*P* = 0.006), 88% (*P* = 0.080) and 213% (*P* = 0.007) for mannose, glucose and glucosamine, respectively. Figure 3 presents examples of the HPLC patterns obtained.

### Global gene expression profile

In order to determine the role of *RLM1* in the biogenesis of *C. albicans* cell wall gene expression profiling analyses on SC5314 and mutant SCRLM1M4A (*Δrlm1/Δrlm1*) were performed in YPD exponential growth phase. The full data set was deposited in the ArrayExpress database from the European Bioinformatics Institute www.ebi.ac.uk/arrayexpress with the accession number E-MEXP-3247.

After filtering, the entire data set resulted in a total of 772 statistically significant differentially regulated ORFs (*P*-value<0.05). Deletion of *RLM1* in *C. albicans* changed the mRNA level of 101 genes with regulation ratios >2.0 (up-regulated and down-regulated; Dataset S1), which is far above the 20 genes identified for *S. cerevisiae Δ*rlm1 mutant grown under the same conditions [26], [38]. Curiously, our data showed that more genes (63.4%) have elevated expression than reduced expression, which is exactly the opposite of what was observed for *S. cerevisiae* by Becerra *et al.* [38], but similar to Jung and Levin [26] results. Gene annotation and classification with the GO terms for *C. albicans* was performed in *Candida* Database. The majority of the up-regulated genes (53.1%) have no known function. The remaining genes (corrected *P*-value<0.05) are involved in cell adhesion related to biofilm formation (*P* = 0.019) and polyamine transport (*P* = 0.037). Regarding down-regulated genes, 48.6% had unknown function, and the ones with significant expression encoded products involved in catalytic activity (43.2%), mainly oxidoreductase activity (10.8%, *P* = 0.026).

Table 1 highlights the genes with known function identified in this study. Genes with higher expression in the mutant than in the wild type strain correspond to genes that are activated due to lack of *RLM1* and may be involved in a compensatory mechanism response [39]. In this study we observed that genes with the highest up-regulation code for proteins involved in cell wall organization and biofilm formation, *ALS1*, *ALS3*, *HWP1*, *ECE1* and *RBT1*, with *ECE1* showing an increase of around 76 fold. *PGA25* that codes for a GPI-anchored protein member of the PGA family was also up-regulated in this study. Members of the PGA family are frequently identified in studies inducing cell wall stress [14] or cell wall regeneration [40]. Genes known to be involved in adaptation to osmotic stress were also up-regulated in this study, such as *GCV2*, *CIT1*, *ENA21* as well as *HGT10*, which codes for a glycerol permease, suggesting that *C. albicans* mutant was under osmotic stress.

**Table 1:** Selected Rlm1p regulated genes.

ORF	Gene name	S.cerevisiae ortholog	Function/Description	Ratio (mutant/wild-type)	Rlm1 binding sequence (location upstream gene sequence)
**Up- regulated genes**					
orf19.3374	*ECE1*	–	Unknown/Hyphal-specific cell wall protein	76	TAWWWWTAGM (−145R)
orf19.1321	*HWP1*	–	Protein binding/Hyphal cell wall protein involved in host defense	37,4	TAWWWWTAGM (−938R)
orf19.1816	*ALS3*	*SAG1*	Protein binding/Adhesin from the ALS family; role in epithelial adhesion, endothelial invasiveness	14,1	CTAWWWWTAG (−0 F; −10R); TAWWWWTAGM (−9 F;−1R)
orf19.5753	*HGT10*	*STL1*	Transporter activity/Glycerol permease involved in glycerol uptake induced by osmotic stress, during cell wall regeneration	13	TAWWWWTAGM (−204F; −976R)
orf19.1327	*RBT1*	*–*	Unknown/Cell wall protein with similarity to Hwp1p	10,4	-
orf19.6336	*PGA25*	*–*	Unknown/Putative GPI-anchored protein	8	TAWWWWTAGM (−316F)
orf19.6078	*POL93*	*YIL080W*	Unknown/nucleic acid binding	7,7	-
orf19.2896	*SOU1*	*SPS19*	Oxidoreductase activity/Enzyme involved in utilization of L-sorbose	6,3	-
orf19.5741	*ALS1*	*SAG1*	Peptide binding/Adhesin; ALS family of cell-surface glycoprotein	6,2	TAWWWWTAGM (−853R)
orf19.3548.1	*WH11*	*HSP12*	Unknown/Protein expressed specifically in white phase yeast-form cells	5,4	TAWWWWTAGM (−96F;−781R)
orf19.4551	*CTN1*	*YAT1*	Transferase activity/Predicted carnitine acetyl transferase	4,9	TAWWWWTAGM (−799R)
orf19.6169	*ATO1*	*ATO2*	Unknown/Putative fungal-specific transmembrane protein	4,6	-
orf19.4393	*CIT1*	*CIT1*	Transferase activity/Protein described as citrate synthase	3,9	-
orf19.4679	*AGP2*	*AGP2*	Transporter activity/Protein described as an amino acid permease	3,8	-
orf19.385	*GCV2*	*GCV2*	Oxidoreductase activity/Glycine decarboxylase P subunit	3,7	TAWWWWTAGM (−213F)
orf19.6139	*FRE7*	*FRE3*	Unknown/Protein similar to ferric reductase Fre10p	3,2	-
orf19.5542	*SAP6*	*BAR1*	Hydrolase activity/Secreted aspartyl proteinase	3,2	-
orf19.4093	*PES1*	*NOP7*	Unknown/Pescadillo homolog required for filament-to-yeast switching	3,2	-
orf19.4211	*FET3*	*FET3*	Oxidoreductase activity/Multicopper oxidas	3,1	CTAWWWWTAG (−784F; −774R); TAWWWWTAGM (−5R)
orf19.1228	*HAP2*	*HAP2*	Unknown/CCAAT-binding factor involved in low-iron response	3	TAWWWWTAGM (−919R)
orf19.3981	*MAL31*	*MAL31*	Transporter activity/Putative high-affinity maltose transporte	2,8	CTAWWWWTAG (−665F; −655R); TAWWWWTAGM (−566R; −897R)
orf19.3265	*TRM1*	*TRM1*	Transferase activity/Protein described as an N2,N2-dimethylguanine tRNA methyltransferase	2,8	-
orf19.2606	*HDA1*	*HDA1*	Hydrolase activity/Histone deacetylase	2,8	-
orf19.2444	*CHS7*	*CHS7*	Protein binding/Protein required for wild-type chitin synthase III activity	2,7	-
orf19.5110	*OPY2*	*OPY2*	Unknown/*S. cerevisiae* ortholog has role in osmosensory signaling pathway, cell cycle arrest	2,6	TAWWWWTAGM (−457F)
orf19.1569	*UTP22*	*UTP22*	Unknown/Putative U3 snoRNP protein involved in rRNA processing	2,6	-
orf19.6948	*CCC1*	*CCC1*	Transporter activity/Putative manganese transporter	2,5	TAWWWWTAGM (−209R)
orf19.5170	*ENA21*	*ENA2*	Unknown/Similar to *S. cerevisiae* sodium transporters	2,5	-
orf19.651	*LYP1*	*LYP1*	Unknown/Putative permease, animo acid transmembrane transporter	2,4	-
orf19.473	*TPO4*	*TPO4*	Transporter activity/Putative sperimidine transporter	2,4	TAWWWWTAGM (−532R)
orf19.5595	*SHE3*	*–*	RNA binding/mRNA-binding protein that localizes specific mRNAs to daughter yeast-form cells and to hyphal tips	2,3	TAWWWWTAGM (−546R)
orf19.5338	*GAL4*	*GAL4*	Transcription regulator activity/Transcription factor involved in control of glycolysis	2,3	-
orf19.4655	*OPT6*	*OPT2*	Transporter activity/Putative oligopeptide transporter	2,3	TAWWWWTAGM (−834F)
orf19.1743	*ACS1*	*ACS1*	Ligase activity/Putative acetyl-CoA synthetase	2,3	-
orf19.6577	*FLU1*	*TPO1*	Transporter activity/Multidrug efflux pump of the plasma membrane	2,2	TAWWWWTAGM (−840F)
orf19.3974	*PUT2*	*PUT2*	Oxidoreductase activity/delta-1-pyrroline-5-carboxylate dehydrogenase	2,2	-
orf19.918	*CDR11*	*PDR5*	Transporter activity/Putative transporter of PDR subfamily of ABC family	2,1	-
orf19.6514	*CUP9*	*CUP9*	Unknown/sequence specific DNA binding	2,1	-
orf19.5071	*NRP1*	*NRP1*	Unknown/Nucleic acid binding	2,1	-
orf19.4815	*YTM1*	*YTM1*	Unknown/Protein similar to *S. cerevisiae* Ytm1p, involved in biogenesis of the large ribosomal subunit	2,1	-
***Down-regulated genes***					
orf19.2525	*LYS12*	*LYS12*	Oxidoreductase activity/mitochondrial homoisocitrate dehydrogenase	−20,1	-
orf19.1868	*RNR22*	*RNR2*	Unknown/ribonucleoside diphosphate reductase	−3,4	-
orf19.866	*RAD32*	*RAD30*	Nucleotidyl transferase activity/Protein similar to *S. cerevisiae* protein with role in nucleotide excision repair	−2,9	-
orf19.7600	*FDH3*	*SFA1*	Oxidoreductase activity/Putative protein of glycine catabolism	−2,9	-
orf19.3749	*IFC3*	*OPT2*	Transporter activity/Oligopeptide transporter	−2,7	-
orf19.2770.1	*SOD1*	*SOD1*	Oxidoreductase activity/Cytosolic copper- and zinc-containing superoxide dismutase	−2,6	-
orf19.5248	*MSO1*	*–*	Unknown/Unknown	−2,5	-
orf19.1756	*GPD1*	*GPD1*	Oxidoreductase activity/Glycerol-3-phosphate dehydrogenase (enzyme of glycerol biosynthesis)	−2,5	-
orf19.5025	*MET3*	*MET3*	Transferase activity/ATP sulfurlyase of sulfate assimilation	−2,4	-
orf19.4777	*DAK2*	*DAK2*	Unknown/Dihydroxyacetone kinase	−2,4	-
orf19.4664	*NAT4*	*NAT4*	Transferase activity/Histone acetyltransferase	−2,4	-
orf19.6640	*TPS1*	*TPS1*	Transferase activity/Trehalose-6-phosphate synthase	−2,3	-
orf19.6116	*GLK4*	*GLK1*	Hexokinase activity/Glucokinase	−2,3	TAWWWWTAGM (−9R)
orf19.5228	*RIB3*	*RIB3*	Lyase activity/3,4-Dihydroxy-2-butanone 4-phosphate synthase	−2,3	TAWWWWTAGM (−756R)
orf19.2341	*HNT1*	*HNT1*	Hydrolase activity/Protein kinase C inhibitor-I	−2,3	-
orf19.5001	*CUP2*	*HAA1*	Unknown/Protein required for normal resistance to copper	−2,2	TAWWWWTAGM (−593R)
orf19.7479	*NTH1*	*NTH1*	Hydrolase activity/Neutral trehalase	−2,1	-
orf19.5000	*CYB2*	*CYB2*	Oxidoredutase activity/Cytochrome b2 precursor protein	−2,1	-
orf19.3359	*ARP8*	*ARP8*	Hydrolase activity/Chromatin-remodeling enzyme complex protein	−2,1	-

Among the up-regulated genes with transporter activity, *AGP2* and *OPT6*, involved in the uptake of amino acids and oligopeptides respectively, were identified in this study. These, together with the action of a secreted protease (*SAP6*), which was also up-regulated, would certainly contribute to the intake of small peptides and amino-acids. The up-regulation of *PUT2* and *GCV2*, whose products are involved in amino acid degradation, may suggest the utilization of alternative carbon or energy sources by *C. albicans* mutant cells. Other genes that could be involved in the interconnection of the pathways required to metabolize non-fermentable carbon sources, i.e. involved in the gluconeogenesis, glyoxylate cycle, and beta-oxidation, are *CIT1*, coding for citrate synthase, *ACS1* an acetyl-CoA synthetase, and *SOU1* a sorbose redutase, which were also up-regulated in this study. Interestingly, *DAK2*, *GLK4*, *GPD1*, *NTH1* and *TPS1*, which products are involved in carbohydrate catabolism appeared as down-regulated (Table 1) and *GAL4*, one of the transcription factors known to be involved in the control of glycolytic enzymes in pathogenic species [41], was up-regulated in our analysis.

Gene expression levels of *ALS3*, *HWP1*, *AGP2*, *PUT2*, *GCV2*, and *CIT1* were checked by quantitative RT-PCR, validating microarrays results (Table S3).

As shown in table 1 only about 35% of the identified ORFs present putative Rlm1p target sequences, suggesting that other target sequences may be recognised by *C. albicans* Rlm1p or that activation of these genes involves the action of other proteins and transcription factors.

### Deletion of *C. albicans RLM1* confers higher in vitro adhesion

**Figure 4:**
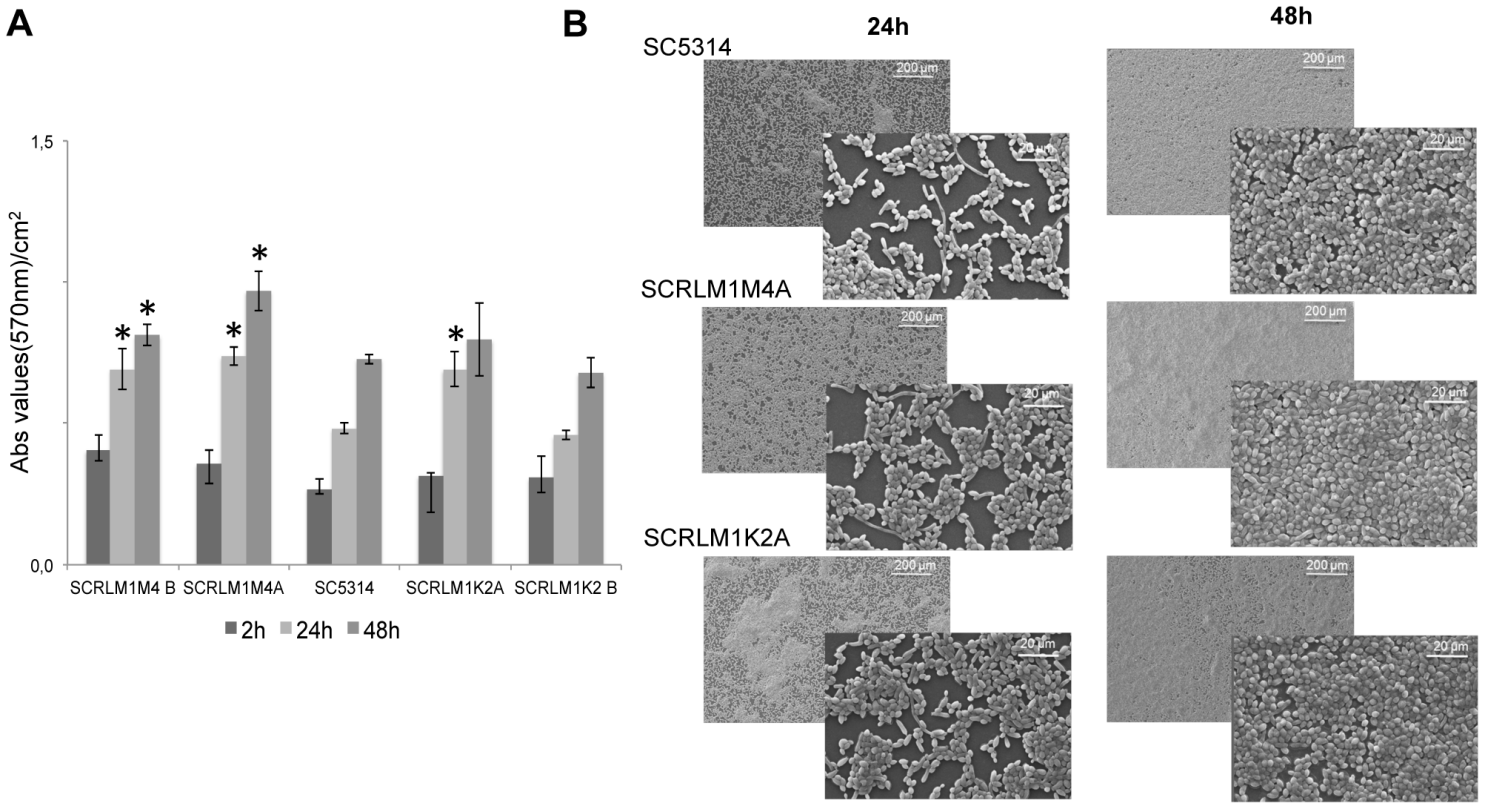
In vitro ability of wild-type, mutant, and complemented strains to adhere to polystyrene surface. (A) Total biomass assessment of wild-type, independent homozygous mutants SCRLM1M4A and SCRLM1M4B (*Δrlm1/Δrlm1*) and complemented strains SCRLM1K2A and SCRLM1K2B (*Δrlm1/Δrlm1+ RLM1*) with crystal violet staining after 2, 24 and 48 hours of incubation; and (B) SEM analysis of adhered cells of wild-type SC5314 strain (*RLM1/RLM1*), the homozygous *rlm1* mutant strain SCRLM1M4A (*Δrlm1/Δrlm1*), and the complemented strain SCRLM1K2A (*Δrlm1/Δrlm1+ RLM1*) after 24 and 48 hours of incubation. The symbol * indicates that measurements were significantly different (*P*<0.005) from the wild-type strain.

Since the proteins that showed higher up-regulation are involved in adhesion and biofilm formation *C. albicans* strains were tested regarding their ability to adhere to a polystyrene surface up to 48 hours. *Candida albicans Δrlm1/Δrlm1* showed a higher ability to adhere to the polystyrene surface compared to the WT and complemented strains, with significant differences (*P*<0.05) seen after 24 h of incubation (Fig. 4A). SEM analyses confirmed the differences in adhesion at 24 and 48 h of incubation (Fig. 4B).

### *C. albicans RLM1* is important for virulence in a murine model of hematogenously disseminated infection

**Figure 5:**
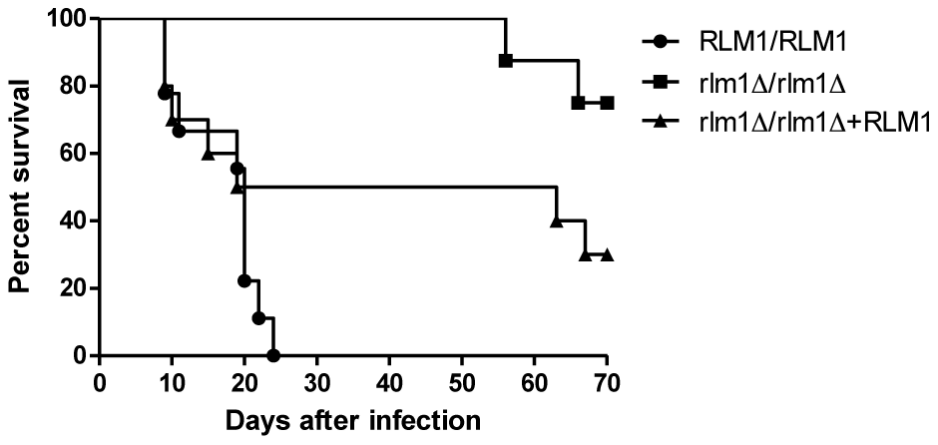
Survival of BALB/c mice following i.v. infection with *C. albicans* strains. Mice were i.v. injected with 5×10^5^ blastospores of wild-type SC5314 strain (RLM1/RLM1), the *rlm1* mutant strain SCRLM1M4A (*Δrlm1/Δrlm1*), and the complemented strain SCRLM1K2A (*Δrlm1/Δrlm1+ RLM1*) and survival was monitored twice daily for 70 days. Results were representative of two independent experiments.

In order to determine if *RLM1* is important for *C. albicans* virulence, BALB/c mice were i.v. injected with 5×10^5^ *C. albicans* cells from wild-type (SC5314), rlm1Δ/rlm1Δ mutant (SCRLM1M4A) or complemented (SCRLM1K2A) strains. All mice injected with SC5314 succumbed to infection within 24 days, presenting a median survival time of 20 days, while 75% of the mice infected with the mutant strain were still alive at the end of the experimental period (70 days) (Fig. 5). Significant differences in the survival time were thus observed between mice infected with SCRLM1M4A strain and SC5314 (*P*<0.0001 by log-rank test). Although mice infected with SCRLM1K2A had a median survival of 41 days, which was much higher than mice infected with WT, 20 days, these differences were not significant (*P* = 0.113) However, median survival time of SCRLM1K2A was significantly shorter than that of mice infected with SCRLM1M4A (*P* = 0.046). These results indicate that the *RLM1* gene is important for *C. albicans* virulence and that introduction of one copy of the gene did not restore completely the virulence phenotype observed in the WT strain.

**Figure 6:**
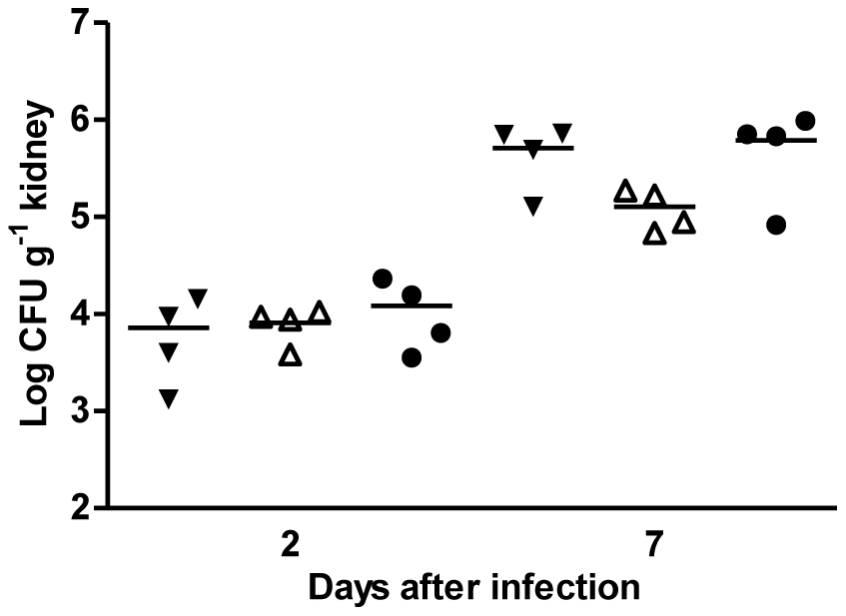
Kidney fungal burden. Groups of four mice injected with 5×10^5^ cells of wild-type SC5314 (▾), mutant SCRLM1M4A (Δ), or complemented SCRLM1K2A (*•*) strains were killed at 2 and 7 days after challenge. Organs were homogeneized in HBSS and the suspension diluted and cultured on Sabouraud dextrose agar. Each symbol represents an individual mouse, and horizontal bars are means of CFU numbers for each group. Results are presented as log of colony-forming units (CFUs).

**Figure 7:**
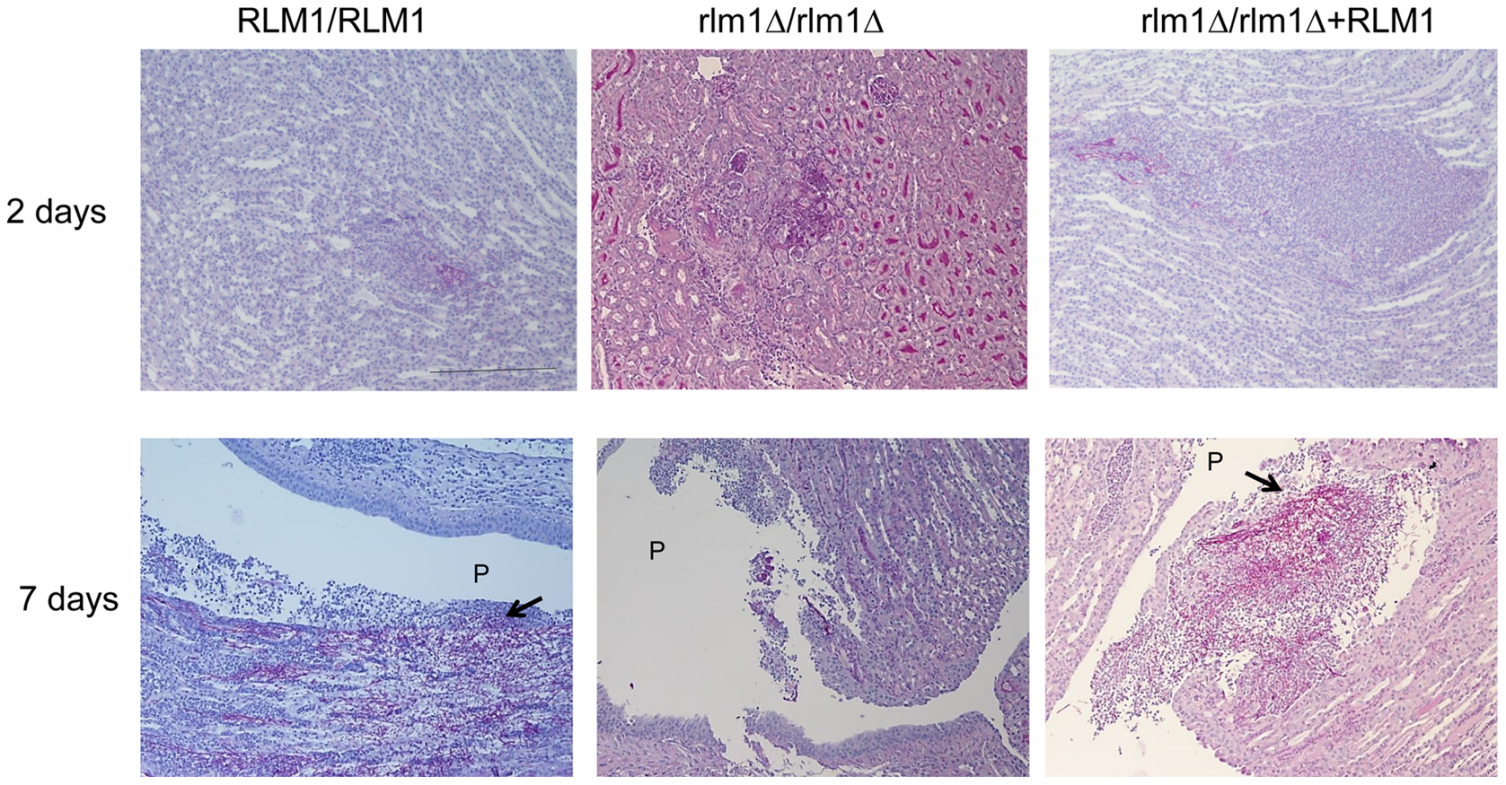
Kidney histology. Representative photomicrographs of HE/PAS-stained paraffin sections of kidneys recovered from BALB/C mice infected with 5×10^5^ cells of wild-type SC5314 (*RLM1/RLM1*), mutant (*Δrlm1/Δrlm1*), and complemented SCRLM1K2A (*Δrlm1/Δrlm1+ RLM1*) *C. albicans* strains at 2 and 7 days post-i.v. infection. Arrows show hyphae invading the pelvis region. P-renal pelvis. Magnification of photographs: 100×. Bar: 100 µm for all photos.

Regarding fungal ability to invade the kidneys all strains tested produced a similar level of infection in these organs after two days of infection (Fig. 6). However, after 7 days of infection, the number of *C. albicans* CFUs in the kidneys of mice infected with the mutant was significantly lower than those of mice infected with the WT and complemented strains (*P*<0.05). Histological analysis of mice kidneys 2 days after infection with strains SC5314 and SCRLM1K2A showed intralesional PAS-positive organisms both in the yeast and hyphal morphology, septated and branched, with moderate multifocal renal medullary interstitial neutrophilic infiltration (Fig. 7). In the kidneys of mice infected with the SCRLM1M4A strain, the fungi appeared as a mixture of ovoid and hyphal cells with a much more restricted leucocytes infiltration. At the latter time point tested, 7 days after infection, analysis of WT-infected and complemented-infected mice showed severe, focally extensive to coalescing, renal medullary interstitial neutrophilic infiltration surrounding numerous PAS positive organisms. These organisms were present mainly as septated, branched hyphal structures, which escaped the medulla and invaded the pelvis region (Fig. 7). In contrast, in kidneys of mice infected with the *rlm1Δ/rlm1Δ* mutant strain, a clear reduction of yeast cells was observed and the remaining hyphal structures were present mainly at the pelvis region, surrounded by neutrophilic infiltration. Invasion of spleen and liver was not consistently seen in all strains (Fig. S1), in agreement with the known higher ability of *C. albicans* to colonize kidneys after mouse systemic infection [43]–[45]. These results confirm that *RLM1* is important for *C. albicans* virulence and capacity to colonize kidneys.

## Discussion

In *S. cerevisiae* the signaling pathway responsible for the CWI is the Slt2 MAP kinase pathway, in which the transcription factor Rlm1 plays a key role in the regulation of genes involved in the maintenance of integrity and cell wall biosynthesis [31], [32]. In *C. albicans* this role is accomplished through the functionally and structurally homologous Mkc1 MAP kinase pathway [10]. Deletions of several *S. cerevisiae* genes involved in the cell wall integrity pathway such as *BCK1*, *MKK1*, *MKK2* or *SLT2/MPK1* confer typical phenotypes, such as failure to grow at elevated temperatures in the absence of an osmostabilizer; sensitivity to caffeine in the medium; failure to grow on glycerol medium; and sensitivity to nitrogen starvation [33]–[37]. However, unlike Mpk1 pathway mutants, *S. cerevisiae Δrlm1* mutant appears to be able to grow normally at elevated temperatures (37°C), grows on glycerol medium and is not sensitive to nitrogen starvation [31]. In this work we studied the role of *C. albicans RLM1* in the biogenesis of cell wall and the consequence of its deletion on the yeast virulence by using a new set of *C. albicans Δrlm1/Δrlm1* mutants constructed using the *SAT1*-flipping strategy [17]. First we compared the same typical phenotypes described for *S. cerevisiae*, performing the tests in parallel with *S. cerevisiae Δrlm1* mutant. We found that *C. albicans Δrlm1/Δrlm1* mutants did not show any of the phenotypes described for *S. cerevisiae* CWI mutants, indicating that *C. albicans* response to these stress factors seem to be largely independent of *RLM1*. However, *C. albicans* mutants were hypersensitive to stresses that affect the cell wall, such as CR and CFW, two well-known cell wall perturbing agents, and CFG which is an inhibitor of the glucan synthesis, reverting the CFG phenotype in the presence an osmostabilizer that confirms the involvement of Ca*RLM1* in the cell wall integrity. Surprisingly, *S. cerevisiae* Δ*rlm1* mutant was insensitive to all these stresses, CR, CFG and CFW, at least under the conditions used in this study, and was hypersensitive to SDS, a detergent known to interfere with the stability of the phospholipid bilayer. The cell wall is important for protection and its polymers share a common path of synthesis, while presenting fungus-specific variations. Sensitivity to CR, CFG and CFW and even SDS has proved to be a powerful tool in revealing cell wall defects, and the differences observed between the two species certainly reflect differences in cell wall composition. *S. cerevisiae* Δ*rlm1* mutant SDS hypersensitivity suggests a loose structure for the cell wall, which allows SDS to reach the plasma membrane and to perturb its organization, leading to cell death much more easily than in *C. albicans*. These observations are in agreement with previous results from Bruno *et al.* [14] that observed a totally different behavior of these mutants in *C. albicans* and *S. cerevisiae*.

It has been described that an increase in the cell wall chitin content is observed after activation of the so-called “compensatory mechanism” in response to the weakening of the cell wall [46]–[48]. In this study, *C. albicans Δrlm1/Δrlm1* mutant showed an increase in the cell wall chitin content in comparison with the wild type strain in cells grown under no stress. Although the microarray analysis did not show over-expression of the genes directly responsible for chitin synthesis, such as *CHS1*, *CHS2*, *CHS3*, or *CHS8* [28], an over-expression of *CHS7* (Chs7p), which is known to be required for the activity of the major *C. albicans* chitin synthase Chs3p [47] was observed. Regarding mannan content none of the genes directly involved in mannosylation [49] were identified in this study, suggesting that the reduction of the mannan content may be indirectly regulated through CaRlm1. These observations indicate that under no stress condition, in the absence of a functional Rlm1p, *C. albicans* cell wall presents a different polymer organization which involves the increase of chitin content and decrease in mannans but does not seem to involve β-1,3-glucans layer. These alterations are essential for the osmotic resistance of the mutant, as it was observed from the microarray data that showed an up-regulation of genes involved in adaptation to osmotic stress. It also suggests that *C. albicans* compensatory mechanism present similarities to the one described for *S. cerevisiae*.

Gene expression analysis performed in cells growing under no stress condition showed a higher number of differentially expressed genes in comparison with the results obtained in a previous work by Bruno *et al.* [14] in which the cells were grown under caspofungin stress. This result suggests that *RLM1* is indeed more committed with the cell wall biogenesis than the remodeling during caspofungin damage. Several genes were identified in the present analysis, including genes involved in the metabolism of carbohydrates like *DAK2*, *GLK4*, *GPD1*, *NTH1* and *TPS1* that were down-regulated. The products of these genes are involved in several pathways that control the utilization of glucose [50], [51], thus we believe that the metabolism of *C. albicans* cells without a functional *RLM1* could be rearranged in order to deviate glucose from utilization as energy source. In fact, the identification of *AGP2*, *OPT6* and *SAP6* as up-regulated genes, may contribute to the intake of di-/tripeptides or amino acids produced by Sap activity [52], [53], implying the utilization of alternative carbon/energy sources. It has been reported that fungal species lacking *GCR1*/2 homologs, like *C. albicans*, have an enrichment of the Gal4p motif in the promoter regions of glycolytic genes [41]. Since *GAL4* was up-regulated in this study it might be possible that the flux of sugars through the glycolytic pathway is even more tightly controlled in *C. albicans Δrlm1/Δrlm1* mutant cells. Blankenship *et al.* [15] based on the transcription of six cell wall damage response genes (*ALS1*, *STP4*, *SOD5*, *DDR48*, *RTA4* and *ECM331*) suggested that many protein kinase (PK) genes could be clustered according to their role in the cell wall regulation. Curiously, in our study the expression pattern of the suggested genes, fits better with the cluster III proposed by Blakenship *et al.* [15] that was implicated in the cell wall biogenesis by regulating the flow of carbohydrates into cell wall biosynthesis pathways, which is exactly what we observe in our study. Therefore we suggest that *RLM1* may be a transcription factor involved in the cell wall biogenesis.

Microarray analysis also showed that the genes with the highest up-regulation in the mutant were *ALS1*, *ALS3*, *HWP1*, *RBT1* and *ECE1*, which are directly involved in the cell wall organization [54]. Furthermore, we observed that the mutant had a higher ability to adhere to polyesterene surfaces than the wild-type and complemented strains. Curiously, Nobile *et al.* [54] described Bcr1 as a transcription factor that governs biofilm formation in an *in vitro* catheter model and showed that *ALS1*, *ECE1*, and *HWP1* are Bcr1 targets. In our study, CaRlm1p behaved as a negative regulator of adhesion in an *in vitro* polysteryne biofilm model, and this mutant presented overexpression of some of the same targets *ALS1*, *HWP1*, and *ECE1*. This observation may suggest that *BCR1* activates genes directly involved in biofilm/adhesion formation, while *RLM1* may regulate negatively the same set of genes.

Finally we detected that the lack of a functional *RLM1* in *C. albicans* reduced the virulence of the mutant strain in the murine model of disseminated candidiasis. This reduced virulence was accompanied by a reduction in the number of CFUs in the kidneys of infected mice. The *Δrlm1/Δrlm1* mutant did not display significant defects in hyphal morphogenesis or overall growth that might account for its attenuated virulence phenotype. Thus, it is plausible that the reduced pathogenicity may be due to the alterations observed in the cell wall composition. The host immune defenses rely on the recognition of conserved molecular patterns in the fungal cell wall, particularly the glucans, which are frequently hidden by the mannoproteins [55]. Defects in cell wall architecture that may expose glucans, during invasive fungal growth, might facilitate the recognition and elimination of *C. albicans* by host immune effector cells. Quantification of *C. albicans Δrlm1/Δrlm1* mutant cell wall polymers showed a decrease in mannans and no difference in the β-glucans layer, which may lead to a higher ability to recognize and eliminate the *Δrlm1/Δrlm1* mutant cells by the host immune system. Curiously, the higher adhesion ability of the mutant could be responsible for the similar kidney's CFUs observed at day two post-infection. However, by day seven, as the mutant cells show a higher susceptibility to immune effector cells they are more easily cleared. Other studies with mutants that affect cell wall organization, including septin organization, also showed decreased virulence in the murine model of disseminated candidiasis, such as *PPZ1*, *PGA13*, *GAL102*, *CDC10*, *CDC11* [49], [56]–[59]. Complementation of *RLM1* partially restored the virulence phenotype, indicating that gene dosage is important. This partial complementation has also been reported for several genes in *C. albicans*, including genes involved in the cell wall integrity [58], [60]–[62].

As a conclusion we hypothesize that the major role for *C. albicans RLM1* may be in the biogenesis of the cell wall, particularly in regulating the flow of carbohydrates into cell wall biosynthesis pathways. Similarly to the compensatory mechanisms described for *S. cerevisiae* cell wall weakening, we also observed that in the absence of *RLM1* and under no stress, *C. albicans* cells present a different cell wall polymer content, which involves chitin and mannan layers, as well as the increase of cell adhesion proteins. This altered cell wall has consequences in the interaction with the environment, increasing adhesion in vitro and reducing virulence in vivo. These results provide a foundation for further mechanistic study of the role of *C. albicans RLM1* in cell wall regulatory responses of this highly successful commensal and opportunistic fungus.

## Supporting Information

Dataset S1 **Microarray data of the genes differentially expressed in the mutant in comparison with the wild-type strain.**
(XLSX)

Figure S1 **Representative spleen (A) and liver (B) sections from mice infected with C. albicans 7 days after challenge.** Spleen presenting red pulp congestion with a great number of neutrophils dispersed on the spleen parenchyma. Liver presenting vascular congestion, small focal mononuclear infiltration and rare neutrophils dispersed on sinusoids (400×, PAS).
(TIF)

Table S1 **Complete genotypes of** ***C. albicans*** **strains used.**
(DOCX)

Table S2 **Oligonucleotide sequences.**
(DOCX)

Table S3 **qRT-PCR expression of** ***ALS3*** **,** ***HWP1*** **,** ***AGP2*** **,** ***PUT2*** **,** ***GCV2*** **, and** ***CIT1*** **.**
(DOCX)
