# Supplementary figures and images for: Participation of Candida albicans Transcription Factor RLM1 in Cell Wall Biogenesis and Virulence
Source: PLoS One. 2014 Jan 23;9(1):e86270. doi: 10.1371/journal.pone.0086270 (PMC3900518; doi:10.1371/journal.pone.0086270)

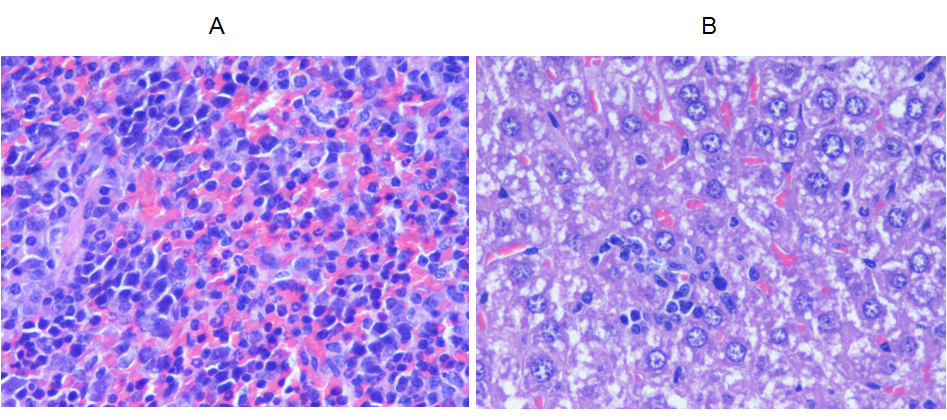

Supplement: Figure S1 — Representative spleen (A) and liver (B) sections from mice infected with C. albicans 7 days after challenge. Spleen presenting red pulp congestion with a great number of neutrophils dispersed on the spleen parenchyma. Liver presenting vascular congestion, small focal mononuclear infiltration and rare neutrophils dispersed on sinusoids (400×, PAS). (TIF) [file pone.0086270.s002.tif]
