# Supplementary material for: Participation of Candida albicans Transcription Factor RLM1 in Cell Wall Biogenesis and Virulence
Source: PLoS One. 2014 Jan 23;9(1):e86270. doi: 10.1371/journal.pone.0086270 (PMC3900518; doi:10.1371/journal.pone.0086270)
Supplement: Table S1 — Complete genotypes of C. albicans strains used. (DOCX) [file pone.0086270.s003.docx]

**Table S1.** *Candida albicans* strains constructed and used in this study.

| **Strain** | **Parental strain** | **Relevant characteristics or genotype*^a^*** | **Source or reference** |
| --- | --- | --- | --- |
| *C. albicans* |  |  |  |
| SC5314 |  | Wild-type *C. albicans* model strain | Gillum et al., 1984 |
| RLM1M1A | SC5314 | *rlm1*Δ::*SAT1-FLIP*/*RLM1* | This study |
| RLM1M1B | SC5314 | *RLM1*/*rlm1*Δ::*SAT1-FLIP* | This study |
| RLM1M2A | RLM1M1A | *rlm1*Δ::*FRT*/*RLM1* | This study |
| RLM1M2B | RLM1M1B | *RLM1/rlm1*Δ::*FRT* | This study |
| RLM1M3A | RLM1M2A | *rlm1*Δ::*FRT*/*rlm1*Δ::*SAT1-FLIP* | This study |
| RLM1M3B | RLM1M2B | *rlm1*Δ::*SAT1-FLIP*/*rlm1*Δ::*FRT* | This study |
| RLM1M4A | RLM1M3A | *rlm1*Δ::*FRT*/*rlm1*Δ::*FRT* | This study |
| RLM1M4B | RLM1M3B | *rlm1*Δ::*FRT*/*rlm1*Δ::*FRT* | This study |
| RLM1K1A | RLM1M4A | *rlm1*Δ::*FRT*/*RLM1-SAT1-FLP* | This study |
| RLM1K1B | RLM1M4B | *RLM1-SAT1-FLP*/*rlm1*Δ::*FRT* | This study |
| RLM1K2A | RLM1K1A | *rlm1*Δ::*FRT*/*RLM1-FRT* | This study |
| RLM1K2B | RLM1K1B | *RLM1-FRT*/ *rlm1*Δ::*FRT* | This study |
| *S. cerevisiae* |  |  |  |
| BY4741 |  | MATa  *his3Δ1 leu2Δ0 met15Δ0 ura3Δ0* | Brachmann *et al*., 1998 |
| YPL089c | BY4741 | MATa *his3Δ1 leu2Δ0 met15Δ0 ura3Δ0 YPL089c::kanMX4* | EUROSCARF |

*^a^ SAT1-FLIP* denotes the *SAT1* flipper cassette
