# Supplementary material for: Participation of Candida albicans Transcription Factor RLM1 in Cell Wall Biogenesis and Virulence
Source: PLoS One. 2014 Jan 23;9(1):e86270. doi: 10.1371/journal.pone.0086270 (PMC3900518; doi:10.1371/journal.pone.0086270)
Supplement: Table S2 — Oligonucleotide sequences. (DOCX) [file pone.0086270.s004.docx]

**Table S2.** Primers used in this study.

| **Primers** | **Sequence (5’- 3’)** |
| --- | --- |
| RLM1-1 | TAGCTGAGCTCAAACCAATATAGTGA |
| RLM1-2 | CGTCTGTCCGCGGTTCTATTTCAATC |
| RLM1-3 | TTCAACTCGAGCAGATGTCAATAAC |
| RLM1-4 | AGTAGGGCCCTAGCCCTAAGTCACG |
| RLM1-compl | TGTCACCGCGGATAAACTAGTCCTTCAT |
| RLM1-CAI | ATGCCATTGAGTGGAATTGG |
| ALS3 | Fw: CGTCCATTTGTTGACGCTTA |
| ALS3 | Rev: GCGGTTAGGATCGAATGGTA |
| HWP1 | Fw: TCTACTGCTCCAGCCACTGA |
| HWP1 | Rev: CCAGCAGGAATTGTTTCCAT |
| AGP2 | Fw: TGTGGCTATGCAGAACTTGG |
| AGP2 | Rev: AGACAGGAACCCCATGACTG |
| PUT2 | Fw: TTCTCCTGGTGTTTGGAACC |
| PUT2 | Rev: TAATGCGGCTGTAGCAGATG |
| GCV2 | Fw: TGGGTGCTGATGTTTGTCAT |
| GCV2 | Rev: AGCTTGTGCTCCCAACATCT |
| CIT1 | Fw: CCACGAAGGTGGTAACGTCT |
| CIT1 | Rev: TTTTTCAATGGCTTCCTTGG |

*^a^* Restriction sites introduced into primers are underlined.
