# Supplementary material for: Participation of Candida albicans Transcription Factor RLM1 in Cell Wall Biogenesis and Virulence
Source: PLoS One. 2014 Jan 23;9(1):e86270. doi: 10.1371/journal.pone.0086270 (PMC3900518; doi:10.1371/journal.pone.0086270)
Supplement: Table S3 — qRT-PCR expression of ALS3 , HWP1 , AGP2 , PUT2 , GCV2 , and CIT1 . (DOCX) [file pone.0086270.s005.docx]

**Table S3.** Relative expression of ALS3, HWP1, AGP2, PUT2, GCV2 and CIT1 genes in the mutant strain SCRLM1M4.

| **Gene** | **Strains** | **Ct** | **ΔCt = target - ref** | **ΔCt mutant - ΔCt WT** | **2exp(ΔΔCt) Fold Change** |
| --- | --- | --- | --- | --- | --- |
| **ALS3** | SC5314 | 24,29 | 9,245 | - | - |
|  | M4 | 22,42 | 6,51 | -2,735 | 6,7 |
| **HWP1** | SC5314 | 27,36 | 12,315 | - | - |
|  | M4 | 23,43 | 7,52 | -4,795 | 27,8 |
| **AGP2** | SC5314 | 24,39 | 9,345 | - | - |
|  | M4 | 22,155 | 6,245 | -3,1 | 8,6 |
| **PUT2** | SC5314 | 23,76 | 8,715 | - | - |
|  | M4 | 22,765 | 6,855 | -1,86 | 3,6 |
| **GCV2** | SC5314 | 22,12 | 7,075 | - | - |
|  | M4 | 20,995 | 5,085 | -1,99 | 4,0 |
| **CIT1** | SC5314 | 22,22 | 7,175 | - | - |
|  | M4 | 21,36 | 5,45 | -1,725 | 3,3 |
| **ACT** | SC5314 | 15,045 | - | - | - |
|  | M4 | 15,91 | - | - | - |
